# Progress in Porcine Kidney Transplantation to Non-Human Primates

**DOI:** 10.3389/ti.2025.14003

**Published:** 2025-02-14

**Authors:** Stéphanie Le Bas-Bernardet, Gilles Blancho

**Affiliations:** ^1^ CHU Nantes, Nantes Université, INSERM, Center for Research in Transplantation and Translational Immunology, UMR 1064, ITUN, Nantes, France; ^2^ CHU Nantes, Nantes Université, Service de Néphrologie et Immunologie Clinique, ITUN, Nantes, France

**Keywords:** xenotransplantation, kidney, genetically modified pigs, nonhuman primates, immunosuppression

## Abstract

Renal xenotransplantation has recently made considerable progress in overcoming the barrier to its use in humans. This progress has been made possible owing to the use of preclinical pig-to-primate models. Overall, renal xenotransplantation has long been associated with lower survival rates than that of porcine hearts (mainly due to its life-sustaining nature). However, the use of the latest strains of genetically modified porcine donors, combined with progress in the control of the anti-porcine immune response and coagulation, has now enabled survival of up to 2 years. Although the pig-to-primate combination has long been considered a perfect reflection of the human situation, it has several limitations, particularly in terms of different natural anti-porcine antibodies. This fact, in association with survival prolongation, which is considered a prerequisite, has led some pioneering teams to cross the line of human application. However, use in humans will remain anecdotal, and further progress in renal xenotransplantation will be difficult to achieve without the use of non-human primates, which will remain complementary, particularly with regard to major innovations that have never been tested in humans.

## Introduction

Significant progress has been made in xenotransplantation (XT) in recent years with the achievement of the first transplantations of genetically modified pig organs into humans.

The evolution of a very long experimental course has involved the use of animal models such as the hamster-to-rat combination, which was very common in the 1990s [1], with the major drawback of being characterized as “concordant” in the absence of preformed antibodies (Abs). Essentially, these earlier models were discordant with pig-to-non-human primate (NHP) (Old World monkey) models, which allow mimicking the pig-to-human situation with the equivalent existence of natural Abs against galactose-α-1,3-galactose (Gal) residues in porcine glycoproteins and glycolipids in NHPs [2].

Through the use of pig-to-NHP models, hyperacute and acute rejection phenomena and their mechanisms have been described. Thus, the first genetically modified porcine organs have been tested and have attracted considerable interest [3]. Among those that were tested, the heart and kidney were the most common organs.

For years, the majority of XT investigators [4, 5] have believed that clinical trials of renal xenotransplantation in humans could be considered once prolonged graft survival with good physiological parameters and low infection and recipient mortality rates were obtained and reasonable immunosuppression was achieved in NHPs.

In this article, we summarize the characteristics of renal XT, focusing not only on the most recent progress achieved in pig-to-NHP models but also on their limitations, with the goal of considering human application to address the issue of organ shortage.

### Latest Developments

#### Survival

For a long time, renal XT researchers have struggled to prolong graft survival, whereas heart survival has progressed from a few months in 2000 [6] to more than 3 years in 2016 [7].

Several characteristics may explain this peculiarity, such as the difference in tissue histology and, most importantly, the sustained nature of the model, which is much more likely to affect the vital prognosis of the recipient.

The use of the first Gal knockout (Gal-KO) pigs as donors helped to prolong survival to almost 3 months in 2 NHP recipients under a specific protocol of tolerance induction with the use of a thymokidney [8]. Finally, Chen et al. reported that this novel generation of genetically modified organs remained subject to Ab-mediated rejection [9], confirming that other xenoantigens (xenoAgs) exist that need to be identified and invalidated.

However, since 2015, considerable progress has been made in terms of the survival time, which has reached more than 2 years [10], mainly because of significant improvements in porcine genome editing and the control of the immune response via immunosuppression.

This long-term survival was achieved by progressive success steps.

Iwase et al. were the first to break the barrier of 100 days of survival, reaching 136 days of survival by using Gal-KO, hCD46, hCD55, hEPCR, hTBM and hCD39 transgenic (Tg) pig kidneys with induction therapy with ATG, rituximab, and cobra venom factor (CVF) and strong maintenance therapy with anti-CD40 monoclonal antibodies (mAbs), rapamycin, steroids, anti-IL6R and anti-TNFR mAbs antiaggregation and anticoagulation [11].

Ma et al. compared groups of pig donors with triple knockout (TKO) with either low or high complement regulatory protein (CRP) expression and immunosuppression based on either CD40 or CD40L blockade and reported survival of up to 316 days in a group of cynomolgus monkeys with TKO/high CRP and CD40L blockade [12].

Kim et al. reported more than 1 year of survival (>400 days) in macaques, with a selection of recipients with low titers of anti-pig Abs and CD4 T-cell depletion associated with anti-CD154-based maintenance [13].

Adams et al. reported survival of more than 500 days in macaques receiving double knockout (DKO) pig kidneys and immunosuppression via C5 and CD154 blockade [14]. Interestingly, in this study, graft survival was inferior with the use of TKO, and the deletion of swine leukocyte antigen (SLA) Class I did not confer any survival benefit.

Recently, while describing the first human decedent models, Anand et al. reported the longest graft survival ever obtained in cynomolgus monkeys, which was more than 2 years (758 days), with the use of TKO, hCD46, hCD55, hTBM, hEPCR, hCD47, hA20 and hHO-1 (TKO-7TG), with or without inactivated porcine endogenous retroviruses (PERVs) [10]. Treatment consisted of T- and B-cell depletion and maintenance therapy with an anti-CD154 mAb, mycophenolate mofetil (MMF), transient tacrolimus and steroids. Interestingly, the authors were able to show a clear benefit of the additional transgenes, as compared with the controls, which had limited survival.

Importantly, these results were obtained mainly in macaques and not in baboons, for which the best XT survival remains more modest, up to approximately 11 months (337 days) [15].

Despite this remarkable progress, it is important to note that this improvement in kidney XT survival may not fully reflect the expected human situation. Indeed, a meta-analysis by Firl et al. comparing the outcomes of 1051 life-sustaining NHP renal allo- and xenotransplants revealed an unexpected minor difference in overall survival between the two situations, with a strict comparison of rhesus monkeys receiving at least 6 months of immunosuppression and Gal-KO organs for XT [16]. Moreover, NHP allotransplant survival appeared to be significantly inferior to that of clinical allotransplants at 6 months, suggesting that clinical renal XT would be better than predicted by preclinical data. This spectacular evolution was due to two main improvements, namely, in genome editing and in immunosuppression, which allowed *in vivo* experiments with NHP models.

#### Impact of the Improvement in Genome Editing: A Proof of Concept in NHPs

Pig-to-NHP XT initially revealed the impact of the first porcine genetic modification via human complement regulatory protein transgenesis to control hyperacute rejection (HAR) [3]. Thereafter, the advantages of invalidating xenoAgs included the ability to control HAR and delay acute humoral xenograft rejection (AHXR) (see articles by *Galli* and by *Wolf* in this Special Issue of *Transplant International*). However, when using this approach, xenoAg expression differed significantly between humans and NHPs, which was a limiting factor for the model (see below).

#### Impact of the Improvement of Immunosuppression

These longer kidney xenograft survival times were further improved by using various new combined immunosuppressive regimens, including the administration of more or less rATG, anti-CD4 and/or anti-CD8 mAbs, anti-CD20 mAbs, anti-CD40 and/or anti-CD154 mAbs, anti-C5 mAbs, MMF, rapamycin or tacrolimus to non-human primates (see article by *Buhler* in this Special Issue of *Transplant International*).

### Lessons From the NHP Model

#### Recipient Selection Based on Low Preformed Anti-Pig IgG Titers

Although the above methods have improved xenograft survival, the presence of anti-pig antibodies in recipients remains an important barrier leading to AHXR.

Similar to humans, NHPs have been shown to have variable levels of anti-pig antibodies that correlate with the occurrence of early AHXR [13, 17, 18].

#### Xenoantigens and Transgene Expression

The generation of KO animals revealed an unexpected appearance of neoantigens that were reactive to natural Abs in NHPs. To assess whether neoantigens associated with multi-transgenic (mTg) TKO cause rejection in the vascularized thymus and kidney xenotransplantation (VT + K XTx) model, Yamada’s group compared in baboons the ability of preformed anti-pig nonGal Abs to bind to Gal-KO PBMCs and TKO and mTg for hCD47, hTBM, hEPCR, HO-1 +/− hCD46 and hCD55 PBMCs. The results revealed that baboons with higher binding titers of preformed IgG to TKO-mTg PBMCs than to Gal-KO PBMCs lost their xenografts due to AHXR at 20 days post-operative days, whereas recipients presenting equivalent binding of preformed Abs to both types of cells maintained their renal function longer without rejection but died of complications [19]. These results suggest that neo-xenoAgs from TKO organs also promote acute xenograft rejection (AXR) [19, 20].

Experiments in NHPs have also revealed that human transgene expression levels may have an impact on graft survival; although donor pigs are clones, they do express variable levels of transgenes with variable phenotypes [21, 22].

#### Physiology and Metabolism of Life-Supporting Pig Kidney Xenotransplants in NHPs

As previously shown by others [23, 24], Anand et al. confirmed sufficient filtration of metabolites by a single transplanted pig kidney in macaques to compensate for the lack of two native kidneys, similar to clinical renal allotransplantation in humans. Furthermore, renal grafts performed with TKO-7TG+/-RI porcine donors supported long-term survival of up to 758 days, in macaques, with parameters such as serum albumin, serum potassium and blood platelet counts remaining within normal ranges in the majority of cases [10].

Proteinuria is known to occur in pigs but is not pathologically significant, unlike in humans and NHPs.

Recent data from pig-to-NHP kidney xenotransplantation revealed moderate proteinuria, probably due to a controlled recipient immune response that reduced physical stress [23]. Kidney graft function was analyzed in baboons cotransplanted with Gal-KO/hCD47 Tg pig kidneys and a vascularized thymus (VT). The results demonstrated that high expression of hCD47 on graft glomerular cells prevented the development of proteinuria. In parallel, the expression of SIRPα (a natural ligand of CD47) was observed in podocytes from porcine naive kidneys and long-term accepted grafts, whereas it was not detected in podocytes from kidney grafts that developed proteinuria [25].

Long-term survivors with functioning grafts had creatinine and potassium levels within normal ranges; however, the phosphorus levels decreased slowly, and serum calcium levels increased [18, 24], confirming data from previous reports [22, 23]. Anemia was observed in recipients of pig-to-NHP kidney xenotransplants and was probably due to the molecular incompatibility between pig erythropoietin (Epo) and the primate Epo receptor [23]. To compensate for the anemia, recipients were treated with recombinant human erythropoietin (rhEpo), with various outcomes. It was shown that long-term survivors treated with rhEpo had stable hematological values, whereas recipients with early rejection developed severe anemia despite the use of rhEpo [18]. However, the occurrence of anemia is still a matter of debate since, in another study, recipients exhibited stable hemoglobin levels without rhEpo treatment [24].

#### Zoonoses and Viral Transmission

Zoonotic disease transmission is a public health issue.

The risk of PERV transmission to NHPs has been constantly monitored in pig kidney-to-NHP XT experiments, and no *in vivo* transmission has ever been detected (see the article by *Denner* in this Special Issue of *Transplant International*). The issue of porcine cytomegalovirus (pCMV) is different because the latter is not believed to infect NHPs or humans. However, the risk of pCMV replication in porcine tissue from CMV-positive donors under strong immunosuppression in recipients is real, as proven by Higginbotham et al. [18] and ourselves. In our experience, pCMV replication has also been detected in the blood of some recipients transplanted with pCMV-positive kidney xenografts, and the majority of these xenografts exhibited early cell nuclear dystrophia, which may be related to viral replication and may have contributed to XT rejection [26].

### NHP Model Limitations

While Tector et al. reported a beneficial effect of cumulative knockout of 3 major xenoAgs (TKO) on human serum reactivity [27], this effect was slightly different in NHPs because, unlike humans, Old World monkeys express N-glycolylneuraminic acid (Neu5Ac) through the persistent expression of the cytidine monophosphate-N-acetylneuraminic acid hydroxylase (*CMAH*) gene, which is responsible for the hydroxylation of Neu5GC [28].

Interestingly, Old World monkeys have increased IgM and IgG binding reactivity to triple-KO pig cells compared with that to double-KO pig cells, which raises the question of the relevance of TKO in NHPs [29].

In the meta-analysis of life-sustaining renal xenotransplantation in NHPs by Firl, the clinical improvement data from wild-type (WT; MST 15 d) to Gal-KO (MST 23 d) and DKO (MST 35 d) support anecdotal reports of decreased survival of TKO (MST 20 d) donor grafts in NHP xenotransplant recipients [16].

However, this feature must be considered with caution as conflicting publications seem to put it into perspective.

Adams reported long-term survival (up to 439 days) in rhesus macaques transplanted with single Gal-KO/CD55 Tg pig kidneys under immunosuppression based on CD40 and complement blockade, which was comparable to the survival time of DKO or TKO pig organs [24]. Finally, the longest survival in primates (>2 years) was obtained with TKO pig kidneys [10]. In the same study, the authors reported that complement-dependent cytotoxicity (CDC) was greater in cynomolgus macaque sera than in human sera [10].

### Regulation and Ethics in Animal Experimentation

Many animal rights organizations oppose the use of animals in research, either pigs or NHPs, because of the risk of disturbing the fundamental species equilibrium, especially at this time of transition to clinical xenotransplantation [22].

Moreover, the cost of experiments with NHPs has recently increased considerably. Taken together, these factors explain why access to these models is now restricted to a very limited number of teams.

## Conclusion

Although pig-to-NHP XT no longer appears to be a perfect research model, it has brought a tremendous amount of knowledge, with impressive prolongation of graft survival with new immunosuppressive regimens, which has allowed recent human clinical application. However, the transfer of XT to humans remains very rare, making statistical analysis based on experimental groups impossible, contrary to NHPs.

Moreover, xenotransplantation experiments in human patients with brain death (a decedent model) as a relevant model [30] have become a matter of debate and cannot be applied easily in Europe.

Pig-to-NHP models of renal xenotransplantation still represent an important experimental opportunity for the study, especially for patients who are not at vital risk, on dialysis or are at a time of desensitization progression.

## Abbreviations

Ab, antibody; AHXR, acute humoral xenograft rejection; AXR, acute xenograft rejection; CMAH, cytidine monophosphate-N-acetylneuraminic acid hydroxylase; CDC, complement-dependent cytotoxicity; CRP, complement regulatory protein; CVF, cobra venom factor; DKO, double knockout; EPCR, endothelial protein C receptor; Gal, galactose-α-1,3-galactose; HAR, hyperacute rejection; mAb, monoclonal antibody; MMF, mycophenolate mofetil; MST, median survival time; mTg, multi-transgenic; Neu5GC, N-glycolylneuraminic acid; NHP, non-human primate; pCMV, porcine cytomegalovirus; PERV, porcine endogenous retrovirus; rATG, recombinant anti-thymoglobulin; rhEpo, recombinant human erythropoietin; SLA, swine leukocyte antigen; TBM, thrombomodulin; Tg, transgene; TKO, triple knockout; TNFR, tumor necrosis factor receptor; XT, xenotransplantation.
